# Assembling a population health management maturity index using a Delphi method

**DOI:** 10.1186/s12913-024-10572-5

**Published:** 2024-01-19

**Authors:** A. F. T. M. van Ede, K. V. Stein, M. A. Bruijnzeels

**Affiliations:** Health Campus The Hague / Department of Public Health and Primary Care, Leiden University Medical Centre, The Hague, The Netherlands

**Keywords:** Population Health Management, Implementation, Maturity, Expert opinion

## Abstract

**Background:**

Although local initiatives commonly express a wish to improve population health and wellbeing using a population health management (PHM) approach, implementation is challenging and existing tools have either a narrow focus or lack transparency. This has created demand for practice-oriented guidance concerning the introduction and requirements of PHM.

**Methods:**

Existing knowledge from scientific literature was combined with expert opinion obtained using an adjusted RAND UCLA appropriateness method, which consisted of six Dutch panels in three Delphi rounds, followed by two rounds of validation by an international panel.

**Results:**

The Dutch panels identified 36 items relevant to PHM, in addition to the 97 items across six elements of PHM derived from scientific literature. Of these 133 items, 101 were considered important and 32 ambiguous. The international panel awarded similar scores for 128 of 133 items, with only 5 items remaining unvalidated. Combining literature and expert opinion gave extra weight and validity to the items.

**Discussion:**

In developing a maturity index to help assess the use and progress of PHM in health regions, input from experts counterbalanced a previous skewedness of item distribution across the PHM elements and the Rainbow Model of Integrated Care (RMIC). Participant expertise also improved our understanding of successful PHM implementation, as well as how the six PHM elements are best constituted in a first iteration of a maturity index. Limitations included the number of participants in some panels and ambiguity of language. Further development should focus on item clarity, adoption in practice and item interconnectedness.

**Conclusion:**

By employing scientific literature enriched with expert opinion, this study provides new insight for both science and practice concerning the composition of PHM elements that influence PHM implementation. This will help guide practices in their quest to implement PHM.

**Supplementary Information:**

The online version contains supplementary material available at 10.1186/s12913-024-10572-5.

## Background

Current developments in the Dutch political landscape increasingly point toward regionalization of healthcare [1]. However, regions and organizations that are willing to change are currently struggling to manage the transition [2]. Population Health Management (PHM) theory provides guidance on how to improve health and wellbeing using a regional approach to sustainable change [3]. The goal of this approach is to accomplish the Quadruple Aim (QA) – improving the experience of care, population health, per capita costs, and the work life of healthcare professionals [4, 5]. In this study, the concept of PHM is used to describe a data-driven approach to improve population health. Using data analytical tools, health and social care data are explored to gain information about the population needs. With the help of health and care professionals and community representatives specific insights are acquired to understand the health needs of different groups of people. Then, context- and population-specific interventions can be developed, implemented, and evaluated to answer to the needs of these people. However, if regions and organizations wish to take action, a purely theoretical knowledge of PHM is insufficient, giving rise to a demand for practice-oriented guidance on how to successfully introduce and manage the process of change to adopt a PHM approach [6, 7].

Based on the small number of successful initiatives and the available literature, it is evident that existing research and tools are insufficient to guide practice in this process of change [8]. There seems to be a divide between practical approaches on the one hand, and pure research, which often overlooks practical implementation, on the other. One example is a review by Steenkamer et al., which represented a step forward in terms of knowledge, but ultimately could only provide a list of theory-based components focussed on unproven strategies [9]. Other research tools used in practice today often have an even narrower focus. This problem is also reflected in the ongoing debate in literature about the relationship between integrated care and PHM. From the author’s perspective, PHM better respects system reform due to its incorporation of a data-driven approach. Current frameworks and tools that support the implementation of integrated care in practice often do not use this data-driven approach and instead focus on a specific subpopulation. The SELFIE framework, for example, supports integrated care programmes for multi-morbidity by ‘describing, developing, implementing and evaluating’ the program [10]. While commendable, this narrow focus on an existing group of patients risks overlooking a population that harbours potential future members of the group. Another example is the SCIROCCO tool, which focuses on the maturity of health systems regarding integrated care and mainly considers improvement of care services [11], resulting in less attention for structural changes to the system. Furthermore, many practice-focused tools are developed within organizations and are therefore neither accessible nor transparent. Examples include the ‘Population Health Maturity Framework’ by Deloitte in the UK, which focuses on care and providers, the ‘Population Health Program Accreditation’ by the NCQA (National Committee for Quality Assurance) in the US, which emphasizes accreditation of health plans, and the ‘Framework for Aligning Sectors’ by the Georgia Health Policy Center in the US, which focuses on aligning sectors to build resilient and equitable communities [12–14]. These tools are not open access and therefore cannot be freely used by new initiatives. Creating a real opportunity for hands-on guidance of regions willing to implement PHM and work towards the QA will require blending of a theoretical understanding of PHM with practical experience of implementation.

To support regions and organizations in PHM implementation and, ultimately, define how the structural changes in the system contribute to improving the QA, we aim to develop a maturity index that unites the theoretical underpinnings of PHM and change management with practical insights from action research. The research described here is an important step in this process. In earlier studies, we combed literature for items that influence the implementation of PHM [15, 16]. In a scoping literature review we divided these items over six PHM elements (accountable regional organization, cross domain business model, integrated data infrastructure, co-designing workforce and community, population health data analysis, and an emergent implementation strategy) and the levels of integration from the RMIC [2, 17], finding a skewed distribution of items across these elements. Building on this earlier work, we now harness expert opinion to expand and validate items that may influence PHM implementation, while addressing three main research questions: *What is the composition of the aforementioned six PHM elements? Which items can be added using practice-based experience? Can expert opinion reduce skewedness in knowledge?*

## Methods

### Study design

To answer these research questions, existing literature formed the basis for expert discussion of items that may influence the implementation of PHM in practice. Due to the discovery in literature of a skewed distribution towards the PHM element ‘accountable regional organisation’, we explicitly focused on the possibility that some items could be missing and the importance of others might have been overstated.

To allow experts to comment on items that reportedly influence the implementation of PHM and discuss the relative importance of these items, we adopted a modified Rand UCLA Delphi panel method [18]. The opinions of Dutch experts was obtained via an online questionnaire (rounds 1 and 2) and an online discussion round (round 3), while the opinions of international experts were solicited via an online questionnaire (round 4) and a physical discussion round (round 5). Although an offline meeting is often preferred, research has shown that online discussions using this method are of equal quality [19].

### Items

Items that reportedly influence the implementation of PHM were previously identified in a scoping review [15, 20] and initially discussed within the research team, leading to a reduction of a 190-item list to a maximum of 20 items per PHM element, numbering 120 in total. This approach was taken to reduce the degree of similarity between items.

### Experts

The RAND UCLA appropriateness method recommends including 7–15 participants in a discussion to ensure sufficient differentiation while preserving opportunities to speak [18]. As it was deemed unlikely that all of those invited would be able to participate, approximately 20 experts per PHM element were initially invited to form six panels (+- 120 experts in total) for the Dutch rounds. Per panel, experts on a range of topics were invited, including those with scientific expertise on a specific element of PHM, with national PHM implementation experience and with local PHM implementation experience, together with experts representing strategic stakeholders. For the international validation rounds, a single panel was formed that discussed all elements of PHM. Approximately 50 international experts with implementation experience in the field of research and policy (variously from Singapore, Canada, Australia, UK, Austria and Ireland) were invited to participate. As we expected more people to respond to an online questionnaire (rounds 1, 2, and 4) than to join a discussion at a specific time (rounds 3 and 5), the study design took into account a decreasing number of participants per round. To ensure participants had seen the complete list of items beforehand, they were only allowed to take part in the discussion round if they had participated in the previous round.

### Rounds

**Fig. 1:**
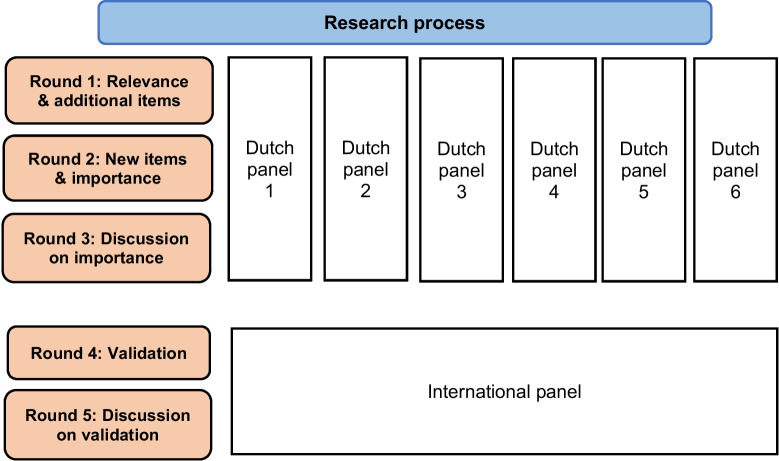
The research process consisted of five rounds and a total of seven panels

Figure 1 shows the complete Delphi process and the various panels. All six panels of Dutch experts completed the same three-round process. In the first round, the experts were asked to fill in an online questionnaire via SurveyMonkey in which they were asked 1) to score items in their PHM element on relevance using a 9-point Likert scale (1 not relevant to 9 very relevant), 2) to place the item on the RMIC, scoring for the level at which the item occurs (system/organizational/professional/clinical) and indicate whether the item qualified as normative or functional. In addition, experts were invited to add any items they felt their PHM element was lacking. In round 2, the experts were again asked to complete an online SurveyMonkey questionnaire in which they answered the same questions as in round 1 but now for the additional items. They were then asked to score all the items on importance and occurrence using a 9-point Likert scale.

Round 3 consisted of an online meeting with a moderated discussion between panel experts that participated in round 2. Prior to the discussion, the experts received a scoring chart detailing their own scoring on round 2, together with agreement scores and median panel scores. The discussion moderators followed a strict protocol, with time limits per item, to ensure that all items with an ambiguous score (agreement < 70% or median score 4–6) could be discussed. The discussion of each item commenced with explanations by the experts who awarded the highest and the lowest scores. After discussion of each item the experts were invited to rescore that item on importance.

The international panel participated in rounds 4 and 5. In round 4 they were asked to fill in an online questionnaire to potentially validate the Dutch panel scores. For each of the PHM elements, they scored agreement as yes or no for all included items and scored all ambiguously scoring items (agreement < 70% or median score 4–6) from round 3 on a 9-point Likert scale of importance. Round 5 took place during an international conference [21], at which a moderated in-person discussion was held to discuss all items that the international panel scored differently with respect to the Dutch panel.

### Data analysis

The criteria of the RAND ULCA appropriateness method were used to analyse the data from each round [18]. In all rounds, for each item the median panel score was defined and divided into three categories: 1–3 low, 4–6 ambiguous, and 7–9 high. When > 70% of experts scored in the same category as the median, agreement was reached. This was also the case for agreement of the RMIC levels in round 1 and 2. If no agreement was reached, the RMIC levels were assigned by one researcher based on the scores and then checked by a second researcher.

In the first round, items were excluded if they reached agreement but had low relevance scores. All other items were advanced to the second round. The comments and items suggested by the experts in round 1 were analysed by two researchers. For each newly suggested item, the researchers first considered whether the item was already present in other PHM elements or if an existing item should be modified. In the second round, items were included if agreement was reached and they scored highly on importance. Low scoring items were excluded. All other items were discussed in round 3. Scores on occurrence of an item supported the discussion of importance in round 3. For items that were neither included or excluded in previous rounds, the round 3 scores were used as the final scores for the Dutch panel.

Round 4 validated all items that received the same scores from the International and Dutch panels. All other items were discussed in round 5 and then re-scored, with these last scores considered the final score from the international panel. When these scores were similar to those of the Dutch panel, the score was considered validated.

## Results

### Experts

Of the 129 Dutch experts that were invited to participate in the first three rounds, 69 completed round 1, 64 completed round 2, and 37 completed round 3. Of the 51 international experts invited to participate in rounds 4 and 5, 23 completed round 4 and 12 completed round 5. Table 1 presents the number of panellists for each round. The participant’s backgrounds were evenly divided between scientific expertise on a PHM element, national implementation experience with a PHM element, local implementation experience with a PHM element and experts representing strategic stakeholders.

**Table 1:** Numbers of participants that participated in the various rounds

	Invited	Round 1	Round 2	Round 3	Invited	Round 4	Round 5
Accountable regional organization	22	12	13	7	51	23	12
Cross-domain business model	25	14	14	7			
Integrated data infrastructure	25	19	13	8			
Co-designing workforce and community	14	7	7	4			
Population health data analytics	23	10	10	6			
Emergent implementation strategy	20	7	11	5			

### Data analysis

**Fig. 2:**
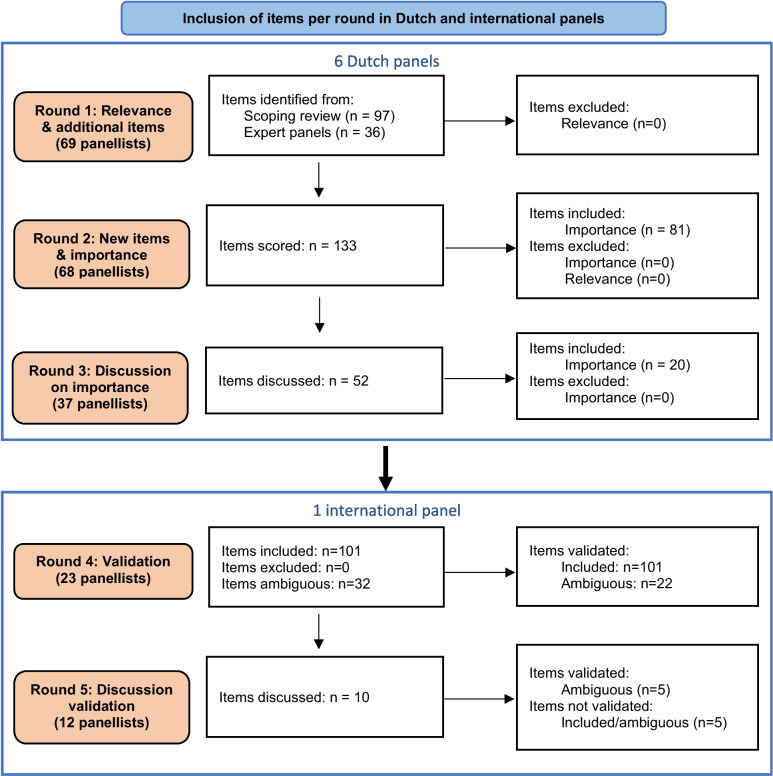
The number of items scored and discussed per round

Figure 2 shows the number of items that were scored and discussed by the panels per round. All items and panel scores per round can be found in the supplementary material (Additional file 1). Reducing the number of items originally identified by the scoping review gave a total of 97 items in round 1. The experts added a total of 36 items across the various PHM elements. No item received a low score during the entire process and therefore no item was excluded. Of 133 items, 32 were scored as ambiguous by the Dutch panels.

### Items not validated

Scores for 123 (101 important/22 ambiguous) of the abovementioned 133 items were validated by the international panel in round 4. Of the ten remaining items, discussed in round 5, five were also deemed ambiguous and thus validated, whereas five items (‘making connections with adjacent domains/sectors’, ‘ability to pool resources at the regional level’, ‘shared standards around data collection’, ‘follow the patient with “real-time” data across the health continuum’, ‘sharing of patient data with professionals’) were considered important by the international panellists but ambiguous by the Dutch panellists and therefore were not validated.

### Items added by the experts

Table 2 shows the number of items added and validated per PHM element. Of the items added by the experts, 12 were scored as ambiguous and 24 scored highly on importance. No specific pattern was found for these additional items, which ranged from functional on the system level (‘adapt laws and regulations if necessary’) to normative on the clinical level (‘trust of the patient in the correct use of the data’). Most items were added within the PHM element ‘integrated data infrastructure’. For the PHM element ‘codesigning workforce and community’ the added items focussed mainly on the relationship between different levels of the RMIC. This was reflected by the items ‘control by local parties (citizens and professionals)’, ‘equity of decision-making between citizens and professionals’, and ‘trust in the co-creation of citizens and professionals’.

**Table 2:** Number of items that were added by the panellists in round 1 and the final score after round 5, divided per PHM element

	Added	Included	Ambiguous	Validated
Accountable regional organization	6	1	5	5
Cross domain business model	4	3	1	4
Integrated data-infrastructure	11	8	3	11
Co-designing workforce and community	5	5	0	5
Population health data analytics	3	2	1	3
Emergent implementation strategy	7	5	2	7

### Distribution of items

Analysis of the remaining gaps in literature and expert opinion consisted of displaying the item distribution across PHM elements and RMIC levels. The research team’s consolidation of items retrieved from the scoping review reduced the total to 97 items. Around half of these items were placed on multiple RMIC levels, resulting in a total of 156 items in the distribution table (Table 3).

**Table 3:** The distribution of items that were derived from literature and presented to the panellists in round 1

	**Accountable regional organisation**		**Cross domain business model**		**Integrated data infrastructure**		**Co-designing workforce and community**		**Population health data analytics**		**Emergent implementation strategy**		**Grand Total**
	**Functional**	**Normative**	**Functional**	**Normative**	**Functional**	**Normative**	**Functional**	**Normative**	**Functional**	**Normative**	**Functional**	**Normative**	
System	3	0	5	5	4	0	3	1	2	0	3	10	36
Organizational	5	10	9	3	4	3	6	2	11	2	5	10	70
Professional	0	5	3	2	3	1	6	4	7	2	3	4	40
Clinical	0	0	2	1	3	0	1	3	0	0	0	0	10
**Grand Total**	**8**	**15**	**19**	**11**	**14**	**4**	**16**	**10**	**20**	**4**	**11**	**24**	**156**

By round 5, 101 items deemed important had been included and validated. Table 4 shows the distribution of these items across PHM elements and RMIC levels. Again, around half of these items were positioned on multiple RMIC levels, resulting in a distribution table with 163 items. Comparing Tables 3 and 4, no significant change was seen in distribution across RMIC levels, but with the addition of expert opinion a more even balance was achieved between the number of normative and functional items. When comparing Table 3 to the initial literature-based distribution table, items also appear more evenly distributed across PHM elements [16]. This shows that the initial process of removing repeated items had the greatest impact on item distribution, and suggests that an excessive focus on ‘accountable regional organization’, as reflected in literature, may lead to crucial aspects of other PHM elements (as identified by experts) being overlooked.

**Table 4:** The distribution of items that were included and validated by round 5

	**Accountable regional organisation**		**Cross domain business model**		**Integrated data infrastructure**		**Co-designing workforce and community**		**Population health data analytics**		**Emergent implementation strategy**		**Grand Total**
	**Functional**	**Normative**	**Functional**	**Normative**	**Functional**	**Normative**	**Functional**	**Normative**	**Functional**	**Normative**	**Functional**	**Normative**	
System	1	0	6	5	7	2	2	2	2	0	5	11	43
Organizational	3	10	8	3	7	5	4	2	9	4	5	11	71
Professional	0	5	3	1	0	1	3	7	6	2	3	5	36
Clinical	0	1	1	1	1	1	1	6	0	0	0	1	13
**Grand Total**	**4**	**16**	**18**	**10**	**15**	**9**	**10**	**17**	**17**	**6**	**13**	**28**	**163**

**Fig. 3:**
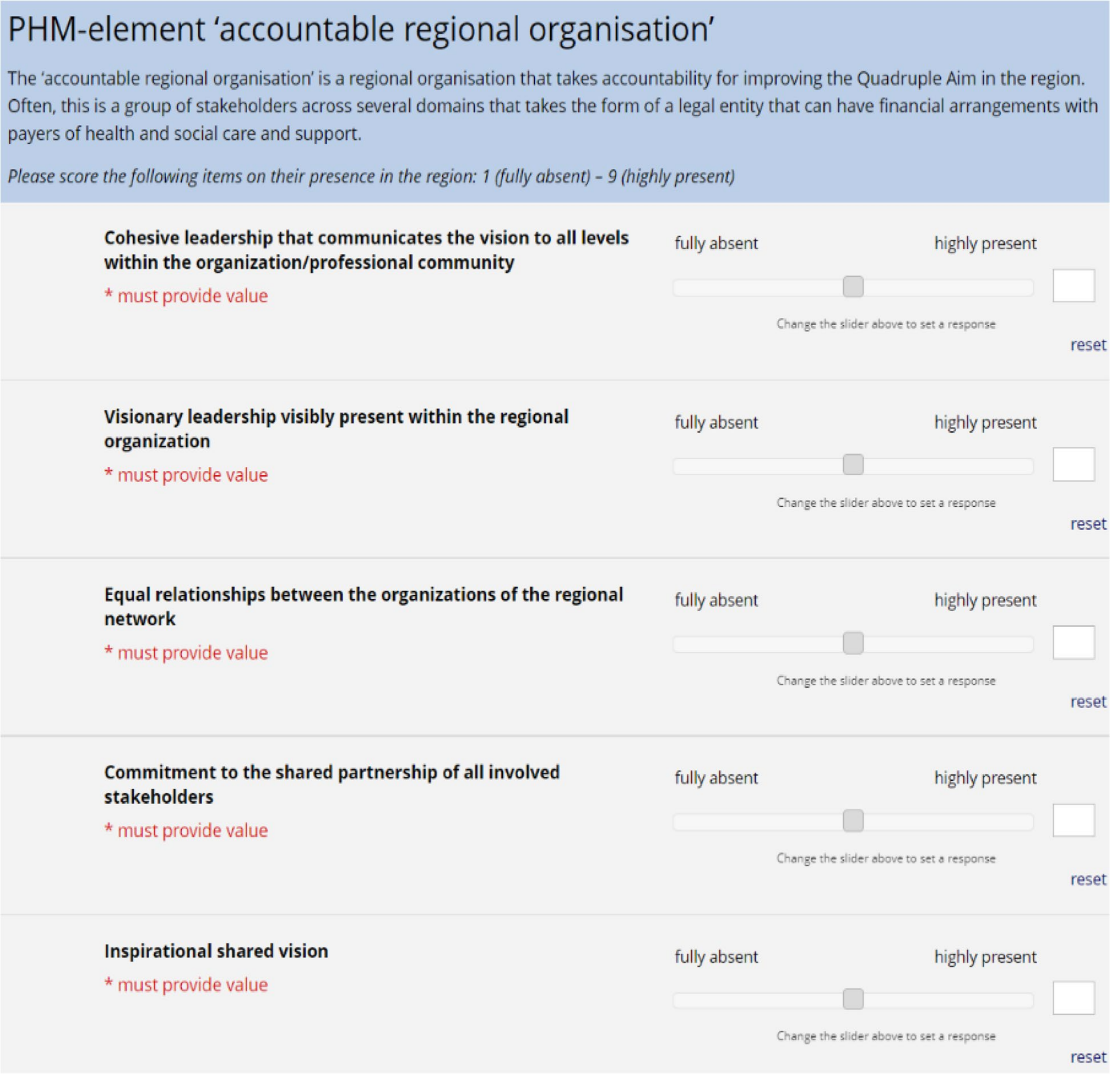
Example of how the first iteration of the PHM-MI might appear when applied in practice

Based on the results of the scoping review and the outcomes of this Delphi study, we could compose a first iteration of a maturity index. Figure 3 shows an example of how this tool might appear when applied in practice, first providing a short explanation of the PHM element and then listing items validated within that PHM element (five are included in the example). The regional presence of each item is scored using a 9-point Likert scale, providing an indicator of regional maturity regarding implementation of PHM.

## Discussion

This study focused on using expert knowledge to identify factors important to the implementation of PHM, to provide better practical guidance for regions willing to adopt PHM. Our results demonstrate that participant expertise can improve understanding of practical issues important to the successful implementation of PHM, and provide insight into how the six PHM elements are best composed in a first iteration of a maturity index. Most index items can be found in current literature, but often appear singular and not connected to PHM. For example, many items from the PHM element ‘accountable regional organization’ occur in studies of cross-sector collaborations, partnerships or governance models [22–25]. Other items, such as ‘evidence-based evaluations with data analysis’, have been specifically addressed in a systematic review [26]. Similarly, items in the PHM element ‘integrated data infrastructure’ were described in a case analysis of a data-linkage project [27]. Our experience and results from literature both suggest that pulling together knowledge from different areas of expertise enhances understanding of the complexity of the implementation challenge [28]. This suggests a broader focus is needed, which was confirmed by our experts who considered all PHM elements important. This conclusion also suggests that earlier frameworks did not adequately encompass the full spectrum. Due to the comprehensive nature of the baseline frameworks, models and expertise applied, the tool developed here can support prioritisation and planning of each step of the transformation journey. It can also indicate regional maturity across the six elements of PHM and identify possible gaps in knowledge and expertise present in the region.

Another aim of the study was to identify possible item additions using practice-based experiences and how these influenced the skewedness of knowledge. Previous literature showed a skewed emphasis on the normative aspects of the PHM element ‘accountable regional organization’ on the regional level and a lack of knowledge on the clinical level, as well as the normative integration of the PHM elements ‘cross domain business model’, ‘integrated data infrastructure’, and ‘population health data analysis’ [16]. By complementing scientific literature with expert knowledge, some of these gaps could be closed and skewedness improved. Another notion is the overlap between the different PHM elements that was confirmed by the experts addition of the items ‘a shared vision for the importance of a data infrastructure’ (in ‘integrated data infrastructure) and ‘cross-disciplinary collaboration around quality of data and analysis’ (in ‘population health data analytics’). Both normative items could have been placed in the PHM element ‘accountable regional organization’ as they specify part of the ‘shared vision’ and ‘cross-disciplinary collaboration. This supports the view that construction of a data infrastructure and data analyses needs to be supported by the organizations involved if the initiative is to be successful [27].

A somewhat unexpected finding was the high degree of validation by the international panel, with only five of 133 items receiving a dissimilar score compared to the Dutch panels. This strongly suggests that the included items are not only important in a Dutch context but are generalizable to many other countries. We therefore hypothesize that the specific context may not be as relevant as generally assumed [29, 30]. This in turn supports the argument that the functionality of the various PHM elements in a PHM initiative are similar across diverse contexts. The context-dependent aspect consists of how this functionality is realized and which form is chosen. This can be illustrated with an example from the PHM element ‘cross domain business model’. The items ‘advance funding is required’ and ‘vision for funding acquisition is supported by everyone’ illustrate the need to actively obtain funds. However, they do not define how or from whom the funds should be obtained. Another contextual aspect that is not specifically displayed in the items is what specific health interventions should be implemented for the different subpopulations in the region. Since every population is different with their own needs, it seems logical that the portfolio of interventions will differ per region. Instead, the items show what is needed to support choosing, funding, implementing and evaluating these health interventions.

Notwithstanding the great potential of the index proposed here, certain results should be interpreted with caution as regards the composition of PHM elements and the impact thereof in practice. One limitation was the number of participants in some panels, for example the small number of experts in the panel on the PHM element ‘codesigning workforce and community’, which in this case was due to difficulty identifying suitable experts. Furthermore, experts on the PHM element ‘codesigning workforce and community’ and experts on ‘emergent implementation strategies’ struggled when scoring items and formulating new items, which also influenced the participation rate of both panels. Another point of caution when interpreting the maturity index is the terminology adopted. While we attempted to adhere as closely as possible to language as derived from literature and used by experts, we should note that maturity index terminology can be ambiguous and items may be open to interpretation due to divergent underlying assumptions. Ambiguity in language is partly attributable to the various disciplines involved, which tend to adopt their own particular language, making it challenging to formulate a framework that is uniformly accepted and understood across all disciplines. Further development of the tool, together with feedback from practice, will help build uniformity and clarity. A practical improvement in clarity might be achieved by developing a glossary, with a brief explanatory text per item. To make the tool more accessible and suitable for implementation in practice, we propose to give end-users and stakeholders a voice in the next phase of tool development. Topics for discussion include usability (for example, web-app development) and practical applications, potentially leading to the inclusion of fewer items. However, this process will have to be closely monitored to avoid losing validity due to insufficient respondent input.

The research presented here can be considered a next step in providing hands-on, evidence-based guidance for regions willing to implement PHM. However, several challenges remain unaddressed. One is the scope of the tool. A 101-item overview across six PHM elements may seem overwhelming for someone unfamiliar with the topic. However, any further shortening of the tool would risk doing an injustice to the broad range of expertise required or the complexity involved. One might even argue that the maturity index should be expanded to include as yet unvalidated or ambiguous items, which were perceived as less important but not unimportant. Although these items might conceivably help guide a region towards success, including all of these items in the maturity index is unlikely to be feasible. Nevertheless, future research should investigate this issue further by adding these items to the framework. Another topic for future research is the question of ‘when to do what’. It can be argued that not all items in the maturity index are of equal importance during the overall implementation process, but that there might be a preferred or logical sequence. However, this sequence might differ between regions according to their maturity on the various PHM elements. Applying the tool in practice in multiple regions will likely help identify gaps and support planning and prioritization, as well as add to our knowledge regarding the context dependency of ‘when to do what’. A third challenge is the connection of these items to an improved QA as outcome [23]. While the items are founded upon issues perceived as important, causality is as yet unproven. Multidisciplinary research that assesses QA improvement over time, alongside scoring of the maturity index, will help inform policy and practice concerning the success of this approach. These considerations will also shape the discussion regarding how the tool could be used, for example as a ‘personality test’ for a region, to allow comparison of regions, or to better inform decision makers concerning the next steps in PHM implementation.

## Conclusion

To conclude, this research informs both science and practice with regard to the composition of PHM elements that influence PHM implementation. This first iteration of a maturity index adds to existing knowledge by providing a scientific foundation and then co-developing the tool with implementers and practitioners. This context, together with its broad scope, sets it apart from other instruments. While most of the items are not new within their area of expertise, they are now innovatively interconnected in a way that will enhance interdisciplinary PHM in practice. This research managed to successfully fuse scientific and practical knowledge of PHM implementation and showed the importance thereof within the complex environment of a transformative change process. As a next step the tool will be applied in practice to determine the best approach to the implementation of PHM for both science and practice.

### Supplementary Information

**Additional file 1.**

## Data Availability

All items and the scores of the panels are included in ‘Additional file 1’. The datasets used and analysed during the current study are available from the corresponding author on reasonable request.

## Abbreviations

PHM: Population Health Management
QA: Quadruple Aim
RMIC: Rainbow model of integrated care
NCQA: National Committee for Quality Assurance
